# The SNOT-22 factorial structure in European patients with chronic rhinosinusitis: new clinical insights

**DOI:** 10.1007/s00405-019-05320-z

**Published:** 2019-02-09

**Authors:** D. Dejaco, D. Riedl, A. Huber, R. Moschen, A. I. Giotakis, L. Bektic-Tadic, T. Steinbichler, P. Kahler, H. Riechelmann

**Affiliations:** 1 Department of Otorhinolaryngology, Head and Neck Surgery, Medical University of Innsbruck, Anichstr. 35, 6020 Innsbruck, Tyrol Austria; 2 University Clinic of Medical Psychology, Medical University of Innsbruck, Speckbacherstr. 23/4, 6020 Innsbruck, Tyrol Austria

**Keywords:** Sinusitis, Paranasal sinus diseases, Nasal polyps, Quality of life

## Abstract

**Purpose:**

The sino-nasal outcomes test-22 (SNOT-22) represents the reference questionnaire to assess patients with chronic rhinosinusitis (CRS). As weak correlations between objective CRS parameters and SNOT-22 total score have been observed, factor analyses have aimed to identify underlying factorial structures. However, ambiguous factor loadings and problematic item-domain assignments have resulted. Moreover, such factor analyses have mainly been performed in non-European CRS patients, while European data remain sparse. This study thus sought to address these issues.

**Methods:**

Principal component analysis and confirmatory factor analysis were performed from SNOT-22 questionnaires completed by European CRS patients. Goodness of fit, internal consistencies, and factor loadings were calculated. Item-domain assignment was based on statistical grounds and clinical meaningfulness. Additionally, this study investigated correlations between SNOT-22 domains and external reference criteria, including Lund–Mackay score, Lund–Naclerio score and the brief symptom inventory 18 (BSI-18).

**Results:**

One hundred and thirty-four European CRS patients were included. Principal component analysis proposed four SNOT-22 domains (“*nasal symptoms*”, “*otologic symptoms*”, “*sleep symptoms*”, “*emotional symptoms*”), which explained 63.6% of variance. Observed item-domain-assignment differed from previously proposed item-domain assignments. All factor loadings were > 0.5, except “cough” (0.42) and “facial pain or pressure” (0.49). For confirmatory factor analysis, satisfactory goodness of fit (RMSEA = 0.66; CFI = 0.92; TLI = 0.90) and internal consistencies (Cronbach-α: total score = 0.93; domains = 0.75–0.91) were observed. Significant positive correlations were found between the “*nasal symptoms*” domain and both the Lund–Mackay score (*r* = 0.48; *p* < 0.001) and the Lund–Naclerio score (*r* = 0.27, *p* < 0.01). Significant positive correlations were also identified between “*emotional symptoms*” and BSI-18 total score (*r* = 0.64, *p* < 0.001).

**Conclusions:**

Principal component analysis performed for SNOT-22 questionnaires completed by European CRS patients indicated a different item-domain-assignment than previously reported. Confirmatory factor analysis suggested acceptable and clinically plausible psychometric properties for the resulting factorial structure. Significant correlations between the “*nasal symptoms*” and the “*emotional symptoms*” domains were observed with objective CRS parameters. The resulting factorial structure with different item-domain assignments may thus be more suitable for European CRS patients.

## Acronyms

AERD: Aspirin-exacerbated respiratory disease
BSI-18: Brief symptom inventory 18
CFI: Comparative fit index
CI: Confidence interval
CRS: Chronic rhinosinusitis
CRSsNP: Chronic rhinosinusitis without nasal polyps
CRSwNP: Chronic rhinosinusitis with nasal polyps
CT: Computed tomography scan
df: Degree of freedom
*e*: Error term
GOF: Goodness of fit
RMSEA: Root mean square error of approximation
SD: Standard deviation
SNOT-22: Sino-nasal outcomes test-22
TLI: Tucker–Lewis index
*χ*^2^: Pearson’s Chi-squared test

## Introduction

To assess health status and health-related quality of life in chronic rhinosinusitis (CRS), the sino-nasal outcomes Test-22 (SNOT-22) has become the reference questionnaire [1]. The SNOT-22 was derived from the Rhinosinusitis Outcomes Measure-31 (RSOM-31), developed by Piccirillo and co-authors [2]. The RSOM-31 aimed to provide a holistic quantification of both health status and health-related quality of life in CRS patients in one total score. The authors used 31 items in seven domains: “nasal symptoms”, “eye symptoms”, “ear symptoms”, “sleep”, “general symptoms”, “practical problems,” and “emotional consequences” [2]. To reduce the complexity of the RSOM-31, abbreviated questionnaires with 16 items [3] and 20 items (SNOT-20) [4] were developed. In the SNOT-20, 11 items from the RSOM-31 were omitted, such as the entire “eye symptoms” domain [4]. However, two cardinal CRS symptoms [5, 6], “stuffed nose” and “difficulty to feel ‘smells’ or ‘tastes’”, were missing. This was reported by Browne and colleagues and consequently supplemented by them [7]. The resulting SNOT-22 was validated by Hopkins and colleagues [1]. Similar to the original RSOM-31, the SNOT-22 provides one total score that measures health status and health-related quality of life in CRS patients [1].

Frequently, only weak correlations between SNOT-22 total scores and objective CRS parameters, i.e., endonasal polyp scores [8–10] or computed tomography (CT) scores [11], have been observed [12–18]. In addition, recent meta-analyses suggested that patient-specific factors may also affect the degree of SNOT-22 change after treatment [19, 20]. To identify the SNOT-22’s underlying factorial structure and thus to reveal hidden correlations, factor analyses have been performed [21–24]. However, these factor analyses have primarily been based on SNOT-22 questionnaires completed by non-European CRS patients [21–24]. A recent study proposed a four-factor solution for the SNOT-22 based on principal component analysis of 177 non-European CRS patients [24]. The extracted principal factors, referred to as “domains”, were named “nasal symptoms”, “otologic symptoms”, “sleep symptoms”, and “emotional symptoms” [24]. However, research during the present study revealed various ambiguous item-domain assignments in this principal component analysis: (1) “facial pain or pressure” was assigned to the “otologic symptoms” domain; this item is suggestive of a cardinal CRS symptom [5, 6], however, and thus may better fit into the “nasal symptoms” domain. (2) “Reduced productivity”, “reduced concentration”, and “frustrated/restless/irritable” were assigned to the “sleep symptoms” domain; these items may be considered cardinal symptoms of depression [25, 26], thus perhaps fitting better into the “emotional symptoms” domain. (3) The most recently added items, the CRS cardinal symptoms “stuffed nose” and “difficulty to feel ‘smells’ or ‘tastes’” [5, 6], could not be attributed clearly to one domain [24]. (4) Additionally, “cough” could not be attributed clearly to one domain (Table 1). The authors state that their item-domain assignment was primarily based on highest factor loadings [24]. However, it is generally recommended that theoretical and empirical information be considered when interpreting statistical results to achieve optimal item-domain assignment [27]. Despite these limitations, the factorial structure proposed by Sedaghat and colleagues [24] was recently validated by confirmatory factor analysis [23]. Good measures of fit and measures of construct validity were reported [23].

**Table 1:** Factor loadings obtained by principal component analysis and confirmatory factor analysis of the present study

Domain	Item number	Item wording	Factor loadings				
			Study (PCA)	Study (CFA)	Sedaghat (PCA)	Feng^a^ (CFA)	Feng^b^ (CFA)
Nasal symptoms							
	1	Need to blow your nose	0.86	0.85	0.85	0.75	0.64
	2	Sneezing	0.63	0.65	0.75	0.67	0.64
	3	Running nose	0.84	0.71	0.80	0.67	0.57
	4	Cough	0.43*	0.74	0.65	0.59	0.57
	5	Post-nasal discharge	0.60	0.47*	0.60	0.73	0.76
	6	Thick nasal discharge	0.77	0.48*	0.70	0.85	0.77
	21	Difficulty to feel ‘smells’ or ‘tastes’	0.51	0.63	0.45*	0.52	0.35*
	22	Stuffed nose	0.76	0.74	0.60	0.89	0.76
Otologic symptoms							
	7	A feeling of full or stuffed ear	0.83	0.80	0.85	0.77	0.72
	8	Dizziness or vertigo	0.77	0.70	0.65	0.83	0.64
	9	Earache	0.82	0.66	0.85	0.71	0.62
	10	**Facial pain or pressure**	**0.49** *	**0.47** *	0.60	0.80	0.84
Sleep symptoms							
	11	Difficulty sleeping	0.89	0.84	0.70	0.74	0.66
	12	Wake up in the middle of the night	0.90	0.87	0.80	0.75	0.62
	13	Lack of a good night’s sleep	0.92	0.95	0.95	0.76	0.65
	14	Wake up tired	0.73	0.73	0.85	0.83	0.77
	15	Fatigued or tired during the day	0.69	0.75	0.80	0.83	0.77
	16	**Reduced productivity**	**0.79**	**0.89**	0.65	0.88	0.75
	17	**Reduced concentration**	**0.79**	**0.78**	0.65	0.90	0.84
	18	**Frustrated, restless or irritated**	**0.81**	**0.84**	0.60	0.92	0.87
Emotional symptoms							
	19	Sadness	0.79	0.53	0.75	0.92	0.87
	20	A feeling of shame	0.50	0.69	0.75	0.67	0.66

For comparison, the previously proposed four-domain structure and its corresponding factor loadings were collected [23, 24]

Bold items loaded more clearly to other domains in the principal component analysis performed in this study as compared to the previously proposed four-domain structure by Sedaghat [24]

*Item with low factor loading < 0.5

^a^Data from Boston cohort as provided by Feng and co-authors [23]

^b^Data from Reno cohort as provided by Feng and co-authors [23]

Apart from factor analyses, correlations with external reference criteria provide an indication of the validity of a proposed factorial structure. Sedaghat’s principal component analysis showed significant correlations between the “nasal symptoms” domain and the Lund–Mackay score (*p* = 0.015), which had not been observed previously using other domains or the SNOT-22 total score [24]. Available data on correlations of the SNOT-22’s “sleep symptoms” and “emotional symptoms” domains with disease-specific questionnaires for depression and sleep disorders remain sparse [28, 29]. Schlosser and colleagues have explored depression-specific outcomes following CRS treatment [29]. Comparing patients without and with depression, they observed worse baseline SNOT-22 total scores [1] and Pittsburgh Sleep Quality Index scores [30] in patients with depression [29]. However, no analysis of the correlation between the domains was performed [29]. Campbell and co-authors explored which patterns of CRS symptoms were associated with lost productivity [28]. Whereas a strong association between the “emotional symptoms” domain and lost productivity was found, no data about its association with depression were reported [28]. Both studies [28, 29] used the two-item patient health questionnaire-2 designed to screen for depression [31].

In short, previously proposed factorial structures for the SNOT-22 may allow a clinically more meaningful interpretation of results [24]. Certain limitations remain, however, several item-domain assignments may be clinically questionable [24], and thus far, there has been no evaluation of the factorial structures from SNOT-22 questionnaires completed by European CRS patients [21–24].

The present study’s aim was to investigate (a) whether ambiguous item-domain assignments based on highest factor loadings [27] would be observed in SNOT-22 questionnaires completed by European CRS patients, and (b) whether item-domain assignment in the relevant items could be based on statistical grounds and clinical meaningfulness [27]. We, therefore, performed (c) a principal component analysis exploring the SNOT-22’s underlying factorial structure and (d) a confirmatory factor analysis-based on this principal component analysis, exploring goodness of fit and measures of construct validity [32]. To evaluate the validity of the resulting factorial structure, correlations with external reference criteria, including the Lund–Mackay score [11], Mackay–Naclerio score [8] and the brief symptom inventory 18 score [33], were evaluated.

## Methods

### Study population

CRS patients were enrolled between 2015 and 2017 at the Department of Otorhinolaryngology, Head and Neck Surgery, Medical University of Innsbruck, Austria. CRS was diagnosed according to established criteria [5, 6]. Patients under 18 years of age and those with allergic fungal rhinosinusitis, fungus ball, sinusitis of dental origin, cystic fibrosis, primary ciliary dyskinesia, immune deficiency, malignancy of the paranasal sinuses or history of alcohol, drug abuse or psychiatric illness were excluded, as were pregnant and lactating women. The local institutional review board approved the study (AN2015-0301).

### Clinical characteristics

Clinical characteristics, including sex, disease duration, atopies [34], asthma [35], aspirin-exacerbated respiratory disease (AERD) [36] and previous endoscopic sinus surgery, were recorded. Means (± standard deviations (SD)) for SNOT-22 total score [1], Lund–Naclerio score [8], Lund–Mackay score [11] and BSI-18 total score [33] were calculated. Data are presented in tabular form for all CRS patients and for CRS patients without nasal polyps (CRSsNP) and CRS patients with nasal polyps (CRSwNP) separately. Chi-squared tests and student’s *t* tests were performed to test for differences between groups (Table 2).

**Table 2:** Clinical characteristics and outcome parameters, with means and standard deviations, for 134 European patients with chronic rhinosinusitis

	CRS (total = 134)	CRSsNP (total = 64)	CRSwNP (total = 70)	*χ* ^2^	*p* value
Sex					
Male	80	36	44	0.61	0.44
Female	54	28	26		
Duration of CRS symptoms (months)					
< 12	27	17	10	13.1	0.01
12–24	25	16	9		
24–60	22	12	10		
> 60	58	18	40		
Comorbidities					
Atopies	67	28	39	1.91	0.17
Asthma	41	13	28	6.10	0.01
Aspirin-exacerbated respiratory disease	22	4	18	9.23	0.002
Previous endoscopic sinus surgery	55	22	33	2.25	0.13
	Mean (± SD)	Mean (± SD)	Mean (± SD)	*t* test	*p* value
Sino-nasal outcome score-22 total score	38.0 (± 20.9)	39.7 (± 21.1)	36.5 (± 20.8)	0.87	0.39
Lund–Mackay score	9.4 (± 5.8)	7.7 (± 4.1)	11.0 (± 6.7)	3.43	0.001
Lund–Naclerio score	3.1 (± 5.8)	n.a.*	n.a.*	n.a.*	n.a.*
Brief symptom inventory 18 total score	9.1 (± 10.9)	11.2(12.8)	7.3(± 8.7)	1.97	0.051

*Not applicable

### Sino-nasal outcome test-22

The SNOT-22 is a validated, self-administered questionnaire that is used to assess CRS patients [1]. It consists of 22 items, rated from 0 (‘no problem at all’) to 5 (‘worst possible symptom’). Possible SNOT-22 total scores range from 0 to 110, with higher SNOT-22 total scores indicating worse symptoms. Good psychometric properties have been reported for the SNOT-22 total score [1]. The current study used the German version of the SNOT-22.

### Lund–Naclerio score and Lund–Mackay score

The Lund–Naclerio score was used for the quantification of nasal polyps [8]. It classifies nasal polyps into four grades: grade 0 (‘absence of polyps’), grade 1 (‘polyps do not prolapse beyond the middle turbinate’), grade 2 (‘polyps prolapse below the middle turbinate’), and grade 3 (‘polyps occlude the entire nasal cavity’) [8]. In CRSwNP patients, Lund–Naclerio scores range from 1 to 6 for both nasal cavities [8]. By definition, CRSsNP patients have a Lund–Naclerio score of 0 [8]. The Lund–Naclerio score was considered the external reference for the SNOT-22 domain “nasal symptoms”.

The Lund–Mackay score was used for quantification of radiologic disease severity as indicated by sinus CT-scans [11]. Extent of radiologic disease is graded from 0 to 2 for each paranasal sinus and for the osteomeatal complex [11]. Total scores range from 0 to 24 [11], with higher scores indicating a greater extent of disease [11]. The Lund–Mackay score is currently considered the radiologic staging system with the highest inter-observer and intra-observer agreement [37]. The Lund–Mackay score was used as the external reference for the SNOT-22 domain “*nasal symptoms*”.

### Brief symptom inventory 18

The BSI-18 is a validated, self-administered questionnaire to assess somatization, anxiety and depression [33]. It consists of 18 items, rated from 0 (‘not applicable at all’) to 4 (‘fully applicable’) [33]. Possible total scores range from 0 to 72 [33]. Higher scores indicate greater distress [33]. In addition to the total score, separate scores on three domains can be calculated: “*depression*”, “*somatization*”, and “*anxiety*” [33]. The BSI-18 was considered the external reference for the SNOT-22 domain “*emotional symptoms*”.

### Statistical analysis

To explore the SNOT-22’s principal factors, referred to as “domains”, an exploratory factor analysis (principal component analysis, Promax) [27] was performed. Scree plots and eigenvalues were used to determine the ideal number of factors [27].

Confirmatory factor analysis [32] was then performed to validate the principal component analysis’ factor solution. To determine the factor solution’s goodness of fit, Pearson’s Chi-squared test (*χ*^2^), degree of freedom (*df*), Tucker–Lewis index (TLI), comparative fit index (CFI), and root mean square error of approximation (RMSEA) with lower and higher bounds of the 95% confidence interval (CI) were calculated [32]. Based on modification indices additional paths between error terms were added to enhance the fit of the model. To maintain parsimony, modification indices were considered only when a substantive basis was present, supported by empirical considerations [27, 32]. Acceptable goodness of fit was defined as RMSEA values of < 0.08 and CFI/TLI values > 0.90 [38, 39]. Finally, domains and external reference criteria were explored for correlations. Although all domains were explored, the Lund–Naclerio score [8] and the Lund–Mackay score [11] were considered the external references for the later-coined domain “*nasal symptoms*”; the BSI-18 [33] served as the reference for the domain “*emotional symptoms*”.

Statistical analyses were performed with SPSS-24 (IBM, Armonk, United States) and the AMOS plugin for confirmatory factor analysis. *P* values < 0.05 were considered significant.

## Results

### Clinical characteristics

One hundred and thirty-four European CRS patients were included. Mean age was 42 ± 17 and ranged from 24 to 79 years. SNOT-22 scores and BSI-18 scores were available for 134 patients, Lund–Naclerio scores and Lund–Mackay scores for 112 patients. Clinical characteristics and means and SD of the outcome parameters are presented in tabular form (Table 2).

### Principal component analysis of the SNOT-22

Principal component analysis was performed to explore the SNOT-22 for potential domains. Bartlett’s test of sphericity (*χ*^2^ (231) = 1638.5, *p* < 0.001) was significant, and the Kaiser–Meyer–Olkin measure verified the sampling adequacy for the analysis (0.89). The initial solution proposed a five-domain structure. Since “cough” loaded to the fifth domain (factor loading 0.56) and significant cross-loading for the second domain (factor loading 0.42) was observed, a four-domain structure was chosen [27]. This provided a more homogeneous item-per-domain distribution. Item-domain assignment was mostly comparable to the previous studies [24]. Domains were named “*nasal symptoms*”, “*otologic symptoms*”, “*sleep symptoms*”, and “*emotional symptoms*”. Eigenvalues indicated that the first four factors explained 41.5%, 10.9%, 5.7%, and 5.5% of variance, respectively, resulting in a total of 63.6% [27]. The diagonals of the anti-image correlation matrix were 0.78–0.92 [27]. All factor loadings were ≥ 0.5, except for “cough” (0.42) and “facial pain or pressure” (0.49). However, for these items, highest factor loadings were observed for “*nasal symptoms*”. Since these items are suggestive of CRS symptoms [5, 6], they were considered plausibly assigned, despite lower factor loading [27] (Table 3).

**Table 3:** Four-domain structure and corresponding factor loadings obtained by principal component analysis of 134 European patients with chronic rhinosinusitis

Item number	Item wording	Domain			
		Nasal symptoms	Otologic symptoms	Sleep symptoms	Emotional symptoms
1	Need to blow your nose	**0.86**	0.36	0.35	0.27
2	Sneezing	**0.63**	0.44	0.18	0.18
3	Running nose	**0.84**	0.25	0.06	0.25
4	Cough*	**0.43**	0.23	0.25	0.17
5	Post-nasal discharger	**0.60**	0.33	0.33	0.41
6	Thick nasal discharger	**0.77**	0.36	0.43	0.27
7	A feeling of full or stuffed ear	0.49	**0.82**	0.45	0.26
8	Dizziness or vertigo	0.14	**0.77**	0.32	0.49
9	Earache	0.25	**0.82**	0.30	0.27
10	Facial pain or pressure^**#**,*^	**0.49**	0.47	0.41	0.35
11	Difficulty sleeping	0.23	0.40	**0.89**	0.46
12	Wake up in the middle of the night	0.27	0.42	**0.90**	0.38
13	Lack of a good night’s sleep	0.35	0.39	**0.92**	0.47
14	Wake up tired	0.36	0.32	**0.73**	0.71
15	Fatigued or tired during the day	0.43	0.41	**0.69**	0.76
16	Reduced productivity	0.46	0.39	0.57	**0.79**
17	Reduced concentration	0.34	0.44	0.37	**0.79**
18	Frustrated, restless or irritated	0.40	0.41	0.52	**0.81**
19	Sadness	0.14	0.34	0.47	**0.79**
20	A feeling of shame	0.22	0.36	0.19	**0.50**
21	Difficulty to feel ‘smells’ or ‘tastes’	**0.51**	0.23	0.33	− 0.09
22	Stuffed nose	**0.76**	0.24	0.43	0.31

Bold items show the highest factor loadings obtained by principal component analysis per domain for each item

*Item with low factor loading < 0.5

^#^Ambiguous item with significant cross-loading

### Confirmatory factor analysis of the identified four-domain SNOT-22 structure

**Fig. 1:**
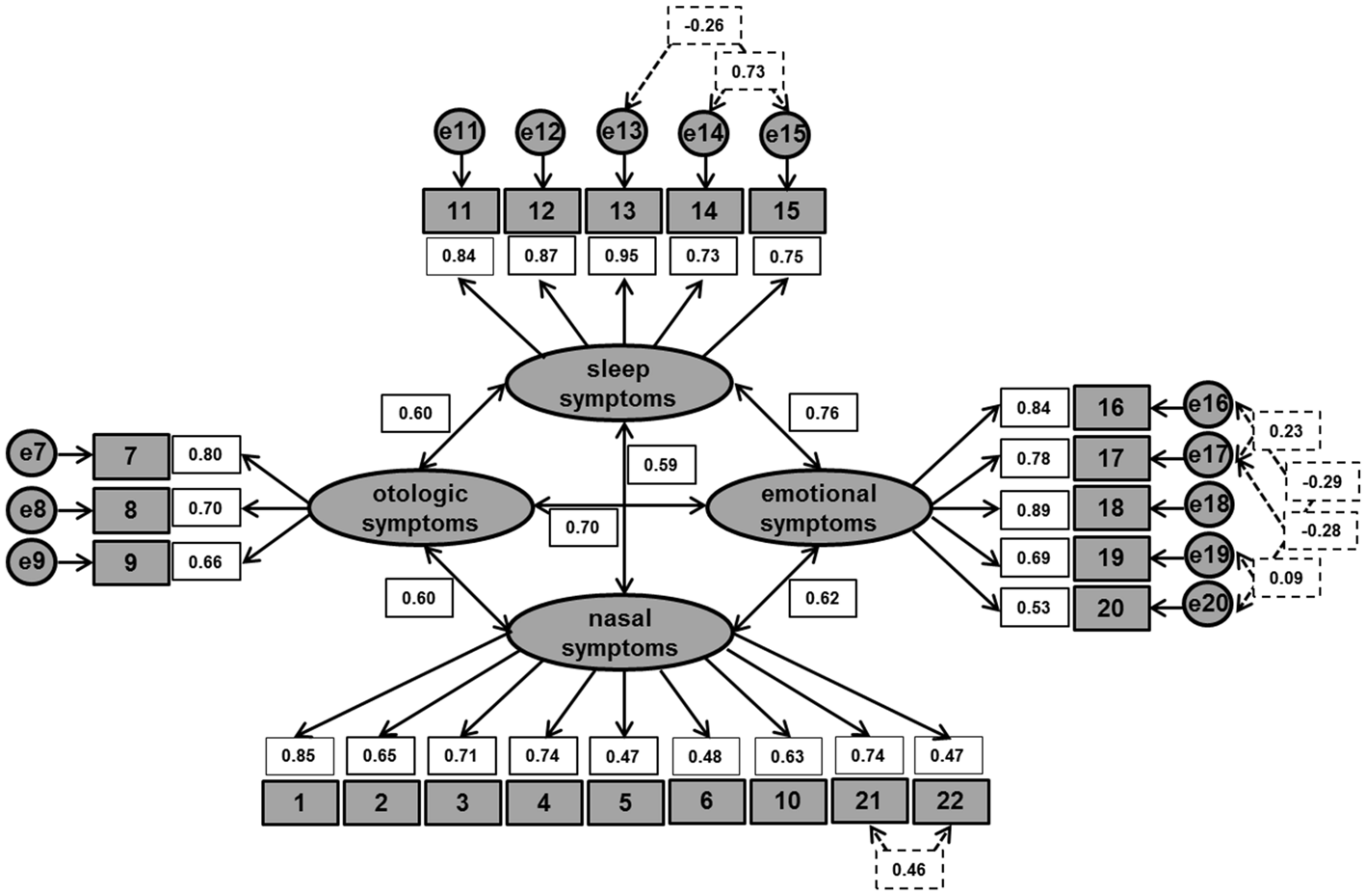
Confirmatory factor analyses of the identified four-domain SNOT-22 structure by principal component analysis, including the four domains “*nasal symptoms*”, “*otologic symptoms*”, “*sleep symptoms*”, and “*emotional symptoms*”. Confirmatory factor analysis was performed with SPSS-24’s AMOS plugin based on the four-domain structure of the principal component analysis. Ellipsoids represent domains, gray rectangles represent SNOT-22 items and their item number, and circles represent error terms. Solid arrows and numeric values in white rectangles represent factor loadings. White rectangles with dashed lines and corresponding dashed arrows represent modification indices. Refer also to the main text and to Table 5

The model used in the confirmatory factor analysis was constructed as suggested by principal component analysis [27] (Table 3). In contrast to the previously proposed four-factor model [23], the item “facial pain or pressure” (item 10) was assigned to the “*nasal symptoms*” domain, while “reduced productivity” (item 16), “reduced concentration” (item 17), and “frustrated/restless/irritable” (item 18) were assigned to the “*emotional symptoms*” domain. Based on the modification indices [32], seven additional paths (depicted below as “↔”) between error terms (depicted below by the letter “e”) were added to improve the model fit [32]. Covaried items included “wake up tired” (item 14) with “lack of a good night’s sleep” (item 13; e13↔e14: *r* = − 0.26) and “fatigued or tired during the day” (item 15; e14↔e15: *r* = 0.73); “reduced productivity” (item 16) with “reduced concentration” (item 17; e16↔e17: *r* = 0.23); “a feeling of shame” (item 20) with “reduced productivity” (e16↔e20: *r* = −0.29), “reduced concentration” (item 17; e17↔e20: *r*= − 0.28) and “sadness” (item 19; e19↔e20: *r* = 0.09); and “difficulty to feel ‘smells or ‘tastes’” with “stuffed nose” (item 22; e21↔e22: *r* = 0.41) (Fig. 1). All correlations between error terms can be explained by similar item wording and content [32]. The inclusion of these additional paths led to a satisfactory model fit. While the χ^2^ was significant (*χ*^2^(196) = 349.97; *p* < 0.001), the RMSEA was below the proposed cut-off of 0.08 (RMSEA = 0.063; 95%CI = 0.048–0.077), and both, CFI and TLI exceeded the cut-off value of 0.90 (CFI = 0.916; TLI = 0.901) (Table 4). All factor loadings were ≥ 0.5, except for “post-nasal discharge” (0.47), “thick nasal discharge” (0.48) and “facial pain or pressure” (0.47). However, for these three items, factor loadings were > 0.35 and, therefore, considered acceptable [32] (Table 5). In terms of internal consistencies for the SNOT-22 total score and the four-domain SNOT-22 structure identified by principal component analysis, Cronbach-alpha for the SNOT-22 total score was 0.93. For the four domains “*nasal symptoms*”, “*otologic symptoms*”, “sleep symptoms”, and “*emotional symptoms*”, Cronbach-alpha was 0.88, 0.75, 0.91, and 0.86, respectively. This means that internal consistencies ranged from “excellent” to “acceptable” [27, 32].

**Table 4:** Fit indices including root mean square error of approximation (RMSEA) with lower and higher bounds of 95% confidence interval (CI), comparative fit index (CFI) and Tucker–Lewis index (TLI) obtained via confirmatory factory analysis [32] to validate the principal component analysis’ [27] factor solution

	RMSEA* (95% CI)	CFI**	TLI***
Fit index	0.063 (0.048–0.077)	0.916	0.901
Level of acceptance [38; 39]	< 0.08	> 0.90	> 0.90

Reference levels of acceptance are provided [38, 39]

*Root mean square error of approximation

**Comparative fit index

***Tucker–Lewis index

**Table 5:** Corresponding factor loadings obtained by confirmatory factor analysis for the emerging four-domain structure obtained by principal component analysis of 134 European patients with chronic rhinosinusitis

Item number	Item wording	Domain			
		Nasal symptoms	Otologic symptoms	Sleep symptoms	Emotional symptoms
1	Need to blow your nose	0.85			
2	Sneezing	0.65			
3	Running nose	0.71			
4	Cough	0.74			
5	Post-nasal discharge*	0.47			
6	Thick nasal discharge*	0.48			
7	A feeling of full or stuffed ear		0.80		
8	Dizziness or vertigo		0.70		
9	Earache		0.66		
10	Facial pain or pressure*	0.47			
11	Difficulty sleeping			0.84	
12	Wake up in the middle of the night			0.87	
13	Lack of a good night’s sleep			0.95	
14	Wake up tired			0.73	
15	Fatigued or tired during the day			0.75	
16	Reduced productivity				0.89
17	Reduced concentration				0.78
18	Frustrated, restless or irritated				0.84
19	Sadness				0.53
20	A feeling of shame				0.69
21	Difficulty to feel ‘smells’ or ‘tastes’	0.63			
22	Stuffed nose	0.74			

*Item with low factor loading < 0.5

### Correlation of the four-domain SNOT-22 structure with Lund–Mackay score [11]

For all CRS patients, the mean Lund–Mackay score [11] was 9.3 ± 5.8. This score was positively correlated with the SNOT-22 total score (*r* = 0.31, *p* < 0.001). The highest correlation was observed for the “*nasal symptoms*” domain (*r* = 0.48, *p* < 0.001). While the Lund–Mackay score [11] was also associated with higher impairment in the “*otologic symptoms*” domain (*r* = 0.27, *p* < 0.01), these correlations were notably smaller. No significant correlation was found between the Lund–Mackay score [11] and the “*sleep symptoms*” (*r* = 0.04, *p* = 0.62) and “*emotional symptoms*” domains (*r* = 0.16, *p* = 0.07).

### Correlation of the four-domain SNOT-22 structure with Lund–Naclerio score [8]

For CRSwNP patients only, the mean Lund–Naclerio score [8] was 1.2 ± 1.7. No significant correlation between the score and the SNOT-22 total score was observed (*r* = 0.20, *p* = 0.13). However, a significant correlation between the “*nasal symptoms*” domain and the Lund–Naclerio score [8] was observed (*r* = 0.40, *p* = 0.003). Neither the “*otologic symptoms*” domain (*r* = 0.10, *p* = 0.48), the “*sleep symptoms*” domain (*r* = − 0.13, *p* = 0.93) nor the “*emotional symptoms*” domain (*r* = 0.09, *p* = 0.54.) correlated significantly with the Lund–Naclerio score [8].

### Correlation of the identified four-domain SNOT-22 structure with BSI-18 [33]

For all CRS patients, the mean BSI-18 score [33] was 9.1 ± 10.9. The SNOT-22 total score was positively correlated with the BSI-18 total score [33] (*r* = 0.64; *p* < 0.001) and its domains “*depression*” (*r* = 0.50, *p* < 0.001), “*somatization*” (*r* = 0.57; *p* < 0.001) and “*anxiety*” (*r* = 0.58; *p* < 0.001). The highest correlation was observed between the BSI-18 total score [33] and the “*emotional symptoms*” domain (*r* = 0.73, *p* < 0.001). While the BSI-18 total score [33] was also associated with the domains “*sleep symptoms*” (*r* = 0.52, *p* < 0.001), “*otologic symptoms*” (*r* = 0.57, *p* < 0.001) and “*nasal symptoms*” (*r* = 0.38, *p* < 0.001), these correlations were considerably smaller.

## Discussion

The SNOT-22 represents the reference questionnaire to assess health status and health-related quality of life in CRS patients in one total score [1]. Frequently, weak correlations between SNOT-22 total score and objective CRS parameters have been observed [12–18]. In addition, recent meta-analyses suggested that patient-specific factors may also affect the degree of SNOT-22 change after treatment [19, 20]. To identify underlying factorial structures and reveal hidden correlations, factor analyses of the SNOT-22 have been performed [21–24], primarily based on four-factor solutions [23, 24]. Although these proposed factorial structures may allow a clinically more meaningful interpretation of results [23, 24], several problems remain: (1) ambiguous factor loadings were observed for certain items [24]; (2) these items were then assigned to domains mainly on statistical grounds rather than based on clinical meaningfulness [24], which (3) resulted in clinically problematic item-domain assignments [24]. Moreover, most previous factorial analyses were conducted on SNOT-22 questionnaires completed by non-European CRS patients [21–24], which may lead to cultural bias. Therefore, this study’s aim was to explore, using principal component analysis [27] and confirmatory factor analysis [32], the factorial structure of SNOT-22 questionnaires completed by European CRS patients. Additionally, the resulting factorial structure was validated with external reference criteria [8, 11, 33].

In principal component analysis, calculated factor loadings represent the correlation of each item with extracted domains. Factor loadings range from 0 to 1. Some items may load high (i.e., close to 1) on one domain and low (i.e., close to 0) on others. Such items can be clearly assigned to one domain. However, items sometimes load intermediately high on two domains, which is referred to as “*cross-loading*”. Item-domain assignment then remains ambiguous. In such cases, assignment should take theoretical and empirical aspects into consideration to achieve optimal item-domain assignment [27].

In the present study, principal component analysis revealed a five-factor solution. However, only “cough” loaded to this fifth domain. A relevant cross-loading was observed for the domain “*nasal symptoms*”. Since “cough” was considered a possible nasal symptom of CRS (i.e., sino-bronchial syndrome) [40], and item-domain assignment to the domain “*nasal symptoms*” resulted in a more homogenous distribution of items/domain, the fifth domain was omitted. “Cough” was assigned to the domain “*nasal symptoms*”. All other principal component analysis factor loadings were > 0.5 except for “facial pain or pressure” (0.49). As “facial pain or pressure” is considered a nasal CRS symptom [5, 6], and factor loading was higher in the “*nasal symptoms*” domain than in the others (0.47, 0.41, and 0.35, respectively), the assignment appeared clinically plausible (Table 3).

Similar weak factor loadings for “cough” (0.57 and 0.59, respectively) have been observed previously [23]. Another frequently weak loading item, “difficulty to feel ‘smells’ or ‘tastes’”, loaded higher than previously reported (0.51 vs. 0.45 and 0.35, respectively) [23, 24]. In contrast to previously proposed four-domain SNOT-22 structures [24], “facial pain or pressure” was assigned to the “*nasal symptoms*” rather than to the “*otologic symptoms*” domain; this was based on factor loading and clinical considerations. Moreover, the items “reduced productivity”, “reduced concentration” and “frustrated/restless/irritable”, suggestive of depression [25, 26], have previously been assigned to the domain “*sleep symptoms*” [24]. In the present study, however, these items loaded more clearly to the domain “*emotional symptoms*”, which appears clinically plausible. Additional details on observed factor loading [23, 24] and previously reported factor loadings are presented in Table 1.

Based on the principal component analysis [27], a confirmatory factor analysis was performed [32], showing acceptable goodness of fit and internal consistencies [38, 39]. All but three items exceeded factor loadings of 0.5. However, since these items loaded acceptable in principal component analysis, and factor loadings were > 0.35, item-domain assignment appeared plausible from clinical and statistical perspectives [27, 32]. Goodness of fit was larger than in the previous studies [23, 24], which may be due to the smaller sample size in our study. Additionally, to maintain parsimony, modification indices were only considered when a substantive basis was present, supported by empirical considerations, as previously recommended [27, 32]. They were limited to a minimum of six modification indices as compared to > 50 in the previous studies [24]. While the extensive use of modification indices may lead to optimal fit, it is not recommended as it may lead to an overestimation of goodness of fit [27, 32].

Most previous studies exploring the correlation between the SNOT-22 total score and the Lund–Mackay score have concluded that there is only little correlation [15, 16]. However, similar to the present study, Brooks and colleagues observed a significant association between preoperative and postoperative SNOT-22 total scores (both *p* < 0.01) driven by the “*nasal symptoms*” domain [13]. In accordance with Sedaghat and co-workers, moreover, a significant correlation was found between the “*nasal symptoms*” domain and the Lund–Mackay score [24]. Although the Lund–Naclerio score [8] has been used in clinical investigations [17], data about correlations with the SNOT-22 total score or its four domains is sparse. In a recent study by Zhang and colleagues exploring endoscopic staging systems, the authors observed no significant correlation between the SNOT-22 total score and the endoscopic polyp score used [17].

In the present study, the domain that differed most from previous investigations was “*emotional symptoms*” [23, 24]. Available data on the correlation of SNOT-22 domains and depression have frequently been based on screening questionnaires [31] with few items, correlating these primarily with the SNOT-22 total score [28, 29]. In the present study, we observed a significant and highly positive correlation between the “*emotional symptoms*” domain and BSI-18 total score [33] (*r* = 0.64, *p* < 0.001). In addition, significant and positive correlations with the previously proposed BSI-18 domains [33] “*depression*”, “*somatization*” and “*anxiety*” were observed (all *r* > 0.50, all *p* < 0.001). Although a significant correlation was also observed for the remaining SNOT-22 domains, these correlations were notably weaker (all *r* > 0.34, all *p* < 0.01). This suggests that the domain “*emotional symptoms*” may also serve as a surrogate for depression, somatization, and anxiety. Nanayakkara and co-authors made similar observations for a comparable questionnaire dealing with depression [41]. The authors observed a strong association between depression and the SNOT-22 total score (*r* = 0.48; *p* < 0.001), the “*emotional symptoms*” (*r* = *0.044; p* < *0.001*) domain and the “*sleep symptoms*” domain (*r* = 0.34; *p* = 0.002) [41]. No association for the “*nasal symptoms*” domain (*r* = 0.18; *p* = 0.18) was observed [41]. The authors recommend the use of at least two domains for the interpretation of the SNOT-22 [41].

Certain limitations apply to the current study. First, the German version of the SNOT-22 has not yet been validated. Although Bauman and colleagues validated the previous version of the SNOT-20 [42], which includes the two previously missing nasal symptoms “stuffed nose” and “difficulty to feel ‘smells or ‘tastes’”, they removed the two items “need to blow your nose” and “lack of a good night’s sleep” [42]. Validation of the complete SNOT-22 for German is clearly required. Second, the number of patients included in this study is small compared to previous studies with up to 800 patients [23]. However, as satisfying psychometric properties [38, 39] were observed for both principal component analysis [27] and confirmatory factor analysis [32], some conclusions may still be drawn.

## Conclusion

The previously proposed four-domain SNOT-22 structure obtained from non-European CRS patients may, despite its limitations, evolve into a reference factorial structure, even for European CRS patients [24]. Principal component analysis performed from SNOT-22 questionnaires completed by European CRS patients suggested a different item-domain assignment, which was supported by confirmatory factor analysis. Moreover, new correlations with external reference criteria were observed. The four-domain SNOT-22 structure observed here is statistically and clinically plausible [38, 39] and perhaps more suitable for European CRS patients. Further studies with international CRS patients should be considered to account for cultural biases.
